# TP53 mutations in functional corticotroph tumors are linked to invasion and worse clinical outcome

**DOI:** 10.1186/s40478-022-01437-1

**Published:** 2022-09-19

**Authors:** Luis Gustavo Perez-Rivas, Julia Simon, Adriana Albani, Sicheng Tang, Sigrun Roeber, Guillaume Assié, Timo Deutschbein, Martin Fassnacht, Monica R. Gadelha, Ad R. Hermus, Günter K. Stalla, Maria A. Tichomirowa, Roman Rotermund, Jörg Flitsch, Michael Buchfelder, Isabella Nasi-Kordhishti, Jürgen Honegger, Jun Thorsteinsdottir, Wolfgang Saeger, Jochen Herms, Martin Reincke, Marily Theodoropoulou

**Affiliations:** 1 Medizinische Klinik und Poliklinik IV, Klinikum der Universität München, Ludwig-Maximilians-Universität München, Munich, Germany; 2 Center for Neuropathology and Prion Research, Ludwig-Maximilians-Universität München, Munich, Germany; 3 Department of Endocrinology, Center for Rare Adrenal Diseases, Assistance Publique-Hôpitaux de Paris, Hôpital Cochin, Paris, France; 4 Université de Paris, Institut Cochin, Inserm U1016, CNRS UMR8104, F-75014 Paris, France; 5 Division of Endocrinology and Diabetes, Department of Internal Medicine I, University Hospital, University of Würzburg, Würzburg, Germany; 6 Medicover Oldenburg MVZ, Oldenburg, Germany; 7 Division of Endocrinology, Hospital Universitário Clementino Fraga Filho, Rio de Janeiro, Brazil; 8 Division of Endocrinology, Department of Internal Medicine, Radboud University Medical Centre, Nijmegen, The Netherlands; 9 Medicover Neuroendocrinology, Munich, Germany; 10 Service d’Endocrinologie, Centre Hospitalier du Nord, Ettelbruck, Luxembourg; 11 Department of Neurosurgery, Universitätskrankenhaus Hamburg-Eppendorf, Hamburg, Germany; 12 Department of Neurosurgery, University of Erlangen-Nürnberg, Erlangen, Germany; 13 Department of Neurosurgery, University of Tübingen, Tübingen, Germany; 14 Neurochirurgische Klinik und Poliklinik, Klinikum der Universität München, Ludwig-Maximilians-Universität München, Munich, Germany; 15 Institute of Neuropathology, University Medical Center Hamburg-Eppendorf, Hamburg, Germany

**Keywords:** Cushing’s disease, Corticotroph, Macroadenomas, *TP53*, *USP8*

## Abstract

Corticotroph macroadenomas are rare but difficult to manage intracranial neoplasms. Mutations in the two Cushing’s disease mutational hotspots *USP8* and *USP48* are less frequent in corticotroph macroadenomas and invasive tumors. There is evidence that *TP53* mutations are not as rare as previously thought in these tumors. The aim of this study was to determine the prevalence of *TP53* mutations in corticotroph tumors, with emphasis on macroadenomas, and their possible association with clinical and tumor characteristics. To this end, the entire *TP53* coding region was sequenced in 86 functional corticotroph tumors (61 *USP8* wild type; 66 macroadenomas) and the clinical characteristics of patients with *TP53* mutant tumors were compared with *TP53/USP8* wild type and *USP8* mutant tumors. We found pathogenic *TP53* variants in 9 corticotroph tumors (all macroadenomas and *USP8* wild type). *TP53* mutant tumors represented 14% of all functional corticotroph macroadenomas and 24% of all invasive tumors, were significantly larger and invasive, and had higher Ki67 indices and Knosp grades compared to wild type tumors. Patients with *TP53* mutant tumors had undergone more therapeutic interventions, including radiation and bilateral adrenalectomy. In conclusion, pathogenic *TP53* variants are more frequent than expected, representing a relevant amount of functional corticotroph macroadenomas and invasive tumors. *TP53* mutations associated with more aggressive tumor features and difficult to manage disease.

**Supplementary Information:**

The online version contains supplementary material available at 10.1186/s40478-022-01437-1.

## Introduction

Pituitary neuroendocrine tumors are the second most common intracranial neoplasm [1]. They are usually benign, but when aggressive they may be particularly difficult to manage, accompanied by high comorbidity and increased mortality [2]. Corticotroph tumors constitute 6–10% of all pituitary tumors, but they represent up to 45% of aggressive pituitary tumors and pituitary carcinomas [2]. Functional corticotroph tumors cause Cushing’s disease (CD), a debilitating condition accompanied by increased morbidity and mortality due to glucocorticoid excess [3]. Pituitary surgery is the first line treatment, but recurrence is observed in 15–20% of cases of whom most are macroadenomas (with a size of ≥ 10 mm) [4]. Treatment options include repeated pituitary surgery, radiation therapy, medical treatment and bilateral adrenalectomy (BADX) [3]. With respect to the latter, corticotroph tumor progression after bilateral adrenalectomy/Nelson’s syndrome (CTP-BADX/NS) is a frequent severe complication and may present with aggressive tumor behavior [5–7].

Corticotroph tumors (including CTP-BADX/NS) carry recurrent somatic mutations in the *USP8* gene in ~ 40–60% of cases [8–13]. These *USP8* mutant tumors are usually found in female patients and are generally less invasive [8–11]. Additional genetic studies identified a second mutational hotspot in the *USP48* gene, but no other driver mutations [14–18]. Focusing on *USP8* wild type corticotroph tumors, we recently discovered *TP53* mutations in 6 out of 18 cases (33%) [17]. Subsequent reports documented *TP53* mutations in small series of mainly aggressive corticotroph tumors and carcinomas [19, 20].

*TP53* is the most commonly mutated gene in malignant neoplasms [21, 22], including brain and neuroendocrine tumors [23, 24]. Until our previous report [17], *TP53* mutations were only described in isolated cases of aggressive pituitary tumors and carcinomas, and were therefore considered very rare events [8, 16, 25–28]. A link between *TP53* mutations and an aggressive corticotroph tumor phenotype has been hypothesized, but the heterogeneity and small size of the studies reported did not support significant clinical associations [17, 19].

To address this, we determined the prevalence of *TP53* variants in a cohort of 86 patients with functional corticotroph tumors, including 61 with *USP8* wild type tumors, and studied the associations between *TP53* mutational status and clinical features.

## Methods

### Patients and samples

We analyzed tumor samples of 86 adult patients: 61 *USP8* wild type and 25 *USP8* mutant. Sixty-six patients (46 females, 20 males) were diagnosed with CD between 1994 and 2020 in Germany (Hamburg, Munich, Erlangen, and Tübingen) and Luxembourg. Twenty additional patients (16 females, 4 males) were diagnosed with CTP-BADX/NS, operated and followed up in 7 different international centers (Nijmegen, Munich, Erlangen, Hamburg, Paris, Rio de Janeiro, and Würzburg). Twenty-three out of 86 samples were collected prospectively between 2018 and 2021, and 63 were retrospective cases (of which 42 were investigated in the context of *USP8* and *USP48* screenings and published elsewhere) [9, 12, 13, 17]. Seventy-one tumors were fresh frozen and 15 were formalin fixed paraffin embedded. Paired blood was available for 12 cases. The median follow-up time after initial diagnosis was 44 months (range 2–384 months).

Endogenous Cushing’s syndrome was diagnosed according to typical clinical signs and symptoms and established biochemical procedures suggesting glucocorticoid excess. Clinical features included central obesity, moon face, buffalo hump, muscle weakness, easy bruising, striae, acne, low-impact bone fractures, mood changes, irregular menstruation, infertility and impotency. Biochemical diagnosis was based on increased 24 h urinary free cortisol (UFC) and late-night salivary cortisol levels, and lack of serum cortisol suppression after low-dose dexamethasone test. A pituitary ACTH source was confirmed by > 2.2 pmol/l (10 pg/ml) basal plasma ACTH, > 50% suppression of serum cortisol during an 8 mg dexamethasone test, and ACTH and cortisol response to corticotrophin releasing hormone stimulation.

The clinical and pathological features of our study cohort are summarized in Additional file 1: Supplementary Table 1. All patients underwent pituitary surgery. The presence of an ACTH-producing pituitary tumor was confirmed histologically after surgical resection. Biochemical remission after surgery was defined as postoperative 24 h-UFC levels below or within the normal range, or serum cortisol levels < 5 µg/dl after low-dose (1 or 2 mg) dexamethasone suppression test. Tumor control was achieved when there was no evidence of regrowth or disease recurrence. Tumor invasion was defined as radiological or intraoperative evidence of tumor within the sphenoid and/or cavernous sinuses [29]. CTP-BADX/NS was defined as an expanding pituitary tumor after bilateral adrenalectomy (BADX) following expert consensus recommendations [5].

### DNA extraction, TP53 amplification and sequencing

Genomic DNA was extracted using the Maxwell Tissue DNA Kit (Promega), Maxwell Blood DNA kit (Promega) or the FFPE DNA mini kit (Qiagen), depending on the type of sample, as described previously [9, 12]. The entire coding sequence of *TP53* (including exons 9β and 9γ) as well as noncoding regions adjacent to each exon were amplified using the GoTaq DNA polymerase (Promega) and specific primers (Additional file 1: Supplementary Table 2). Amplification of *USP8* hotspot region and Sanger sequencing were performed as described previously [9, 12]. Chromatograms were analyzed using the Mutation Surveyor v4.0.9 (Soft Genetics). Samples were examined for *TP53* coding and splicing variants. Variant position and pathogenicity was investigated in ENSEMBL (www.ensembl.org), the UCSC Genome Browser (http://genome-euro.ucsc.edu), the IARC *TP53* database (https://p53.iarc.fr/TP53GeneVariations.aspx), the Catalogue Of Somatic Mutations in Cancer (COSMIC; https://cancer.sanger.ac.uk/cosmic), ClinVar (https://www.ncbi.nlm.nih.gov/clinvar/), PHANTM (http://mutantp53.broadinstitute.org/), the Human Splicing Finder (HSF; http://www.umd.be/HSF3/) and VarSEAK splicing predictor (https://varseak.bio/). Variant frequencies on the general population were obtained from the Allele Frequency Aggregator (ALFA) project [30], the Genome Aggregation Database (gnomAD) [31] and the International Genome Sample Resource 1000Genome project [32]. Throughout the text, variants refer to NC_000017.11 (genomic DNA), ENST00000269305.9 (coding DNA) and ENSP00000269305.4 (protein), following the Human Genome Variation Society (HGVS) standard nomenclature system.

### Statistical analysis

Statistical analysis was performed with the software package SPSS v24 (IBM). We used t-test or one-way ANOVA to analyze the association of *TP53* variants with age, body mass index; Mann–Whitney U and Kruskal–Wallis to test non-parametric variables, such as tumor size, hormone levels, Ki67 index and p53 score. We corrected the analysis for multiple comparisons with the Bonferroni test. Categorical variables were analyzed using a chi-square test or Fisher exact test when needed. Survival analysis was performed using Kaplan–Meier curves with log-rank tests, and multivariate Cox regression. An exact, two-tailed significance level of *P* < 0.05 was considered to be statistically significant.

## Results

### Analysis of TP53 nucleotide variants

We analyzed all *TP53* coding exons (including exons 9β and 9γ) and adjacent intronic noncoding sequences in 61 *USP8* wild type tumors (49 CD and 12 CTP-BADX/NS). Of these, 13 were microadenomas (< 10 mm) and 48 macroadenomas (≥ 10 mm) at the time of the current operation. A separate group of 25 *USP8* mutant tumors (17 CD and 8 CTP-BADX/NS) that were mainly macroadenomas (n = 19) was used for multiple comparison.

We found 59 variants in our cohort: 30 exclusively in *USP8* wild type, 21 in *USP8* mutant, and 8 in wild type and mutant tumors regardless of *USP8* mutational status. No indels in the coding region of *TP53* were detected. In addition, we did not find any genetic variant affecting *TP53* splicing.

Nine out of 30 variants found in *USP8* wild type tumors were either reported in the COSMIC database as pathogenic or absent from the common variant databases (1000Genomes, gnomAD, ALPHA) or had allele frequency < 0.0001. They were all described in cancer series: 5 as pathogenic or likely pathogenic in ClinVar, 2 as variants of uncertain significance (VUS) and 2 were not described in ClinVar (Table 1). All variants are reported to alter protein function and show clear loss of transactivation activity in a yeast based assay (Table 1) [33].

**Table 1:** Functionally relevant *TP53* variants found in 9/86 corticotroph tumors

#	Genomic position^a^	Coding sequence^b^	Protein^c^	Varian type	Exon	Domain	ClinVar ID Interpretation	COSMIC ID FATHMM prediction (score)	% transcriptional activity^d^
1	g.7675214A > T	c.398 T > A	p.Met133Lys	Missense	5	DNA-binding	NA	COSV52821486 Pathogenic (1.00)	11.33%
2	g.7674887C > T	c.644G > A	p.Ser215Asn	Missense	6	DNA-binding	376662 Likely pathogenic^e^	COSV52686793 Pathogenic (0.99)	12.96%
3	g.7674249A > T	c.714 T > A	p.Cys238Stop	Missense	7	DNA-binding	NA	COSV52840491 Pathogenic (0.90)	NA
4	g.7674245 T > C	c.718A > G	p.Ser240Gly	Missense	7	DNA-binding	584921 Likely pathogenic^e^	COSV52677032 Pathogenic (0.96)	29.89%
	g.7674190 T > G	c.773A > C	p.Glu258Ala	Missense	7	DNA-binding	458563 VUS	COSV52688395 Pathogenic (0.99)	0.08%
5	g.7674217C > G	c.746G > C	p.Arg249Thr	Missense	7	DNA-binding	376015 Pathogenic	COSV52697169 Pathogenic (0.99)	0.31%
6, 7	g.7673802C > T	c.818G > A	p.Arg273His	Missense	8	DNA-binding	12366 Pathogenic	COSV52660980 Pathogenic (1.00)	2.51%
8	g.7670700G > C	c.1009C > G	p.Arg337Gly	Missense	10	Tetramerization	237938 VUS	COSV52816817 Pathogenic (0.84)	10.08%
9	g.7670678A > G	c.1031 T > C	p.Leu344Pro	Missense	10	Tetramerization	12375 Likely pathogenic​	COSV52687285 Pathogenic (0.99)	11.44%

^a, b, c^ Reference sequence identifiers GRCh38/hg38 NC_000017.11 (genomic), ENST00000269305.9. (transcript) and ENSP00000269305.4 (protein), respectively

^d^ As measured by [33, 34]

^e^ Evidence in favor of likely pathogenic variant in sporadic cancer and/or hereditary cancer-predisposing syndrome; VUS or conflicting interpretation in Li-Fraumeni syndrome

Seven variants target amino acids within the DNA-binding domain, essential for p53 activity, disrupting S2’ and S7 β-sheets or the L3 loop spatial conformation. The other two [c.1009C > G (p.Arg337Gly) and c.1031 T > C (p.Leu344Pro)] locate in the tetramerization domain and keep p53 protein as monomer impairing its transactivation activity [34]. From the 9 variants, 8 affect highly conserved p53 residues, while in c.1031 T > C (p.Met133Lys) the methionine alternates with leucine or valine among species. This variant alters protein folding, probably reducing DNA affinity [35], while the substitution of a methionine that acts as an alternative start codon abolishes the transcription of isoforms ∆133p53α, ∆133p53β and ∆133p53γ. The 9 variants were detected in nine cases (henceforth referred to as *TP53* mutant; Table 1). Two tumors from unrelated patients (#6 and #7) carried the same variant c.818G > A (p.Arg273His), while one tumor (#4) carried two variants (c.718A > G and c.773A > C). Seven variants were found in heterozygosis, while the other two (from patients #1 and #2) in homozygosis. From these two, we only had paired blood/tumor samples from patient #1 and detected the variant only on the tumor sample, indicative of loss of heterozygosity (Additional file 1: Supplementary Fig. 1A). Similarly, we could demonstrate the somatic origin of the *TP53* variants in four other patients with paired tumor/blood samples (#3, #5, #6 and #9).

The remaining 21/30 variants found in *USP8* wild type and all 21 variants found in the *USP8* mutant tumors were described as benign, likely benign or VUS with no evidence of affecting protein function. All tumors with these variants were considered *TP53* wild type. From the 21 variants found in the *USP8* wild type tumors (henceforth referred to as *TP53/USP8* wild type group), 7 were non-synonymous variants, 8 synonymous variants and 6 non-coding variants without splicing effect. From the 21 variants found in the 25 *USP8* mutant tumors, nine were synonymous, four non-synonymous and eight non-coding without splicing effect. In addition, eight variants were found in tumors regardless of *USP8* mutational status that were not categorized as *TP53* mutations. The intronic variant c.782 + 62G > A was found in heterozygosis in 6/70 samples. It was not reported in any database and is not predicted to have any splicing effect. The remaining seven are common variants classified as benign or likely benign in ClinVar and their allele frequencies were similar to those reported for the general population (ALFA, gnomAD and 1000Genome project) (Additional file 1: Supplementary Table 3).

Summarizing, all *TP53* mutations were found in the *USP8* wild type tumors, leading to a prevalence of 15% in this subgroup.

### Clinical presentation of patients with TP53 mutant tumors

Patients with *TP53* mutant tumors (n = 9) tended to be diagnosed at older age compared to *TP53/USP8* wild type tumors (n = 52) (t-test *P* = 0.069; Table 2). This was significant after including the *USP8* mutant group (n = 25) in the multiple comparison analysis (ANOVA *P* = 0.024, Table 2) and when *TP53/USP8* wild type and *USP8* mutant tumors were combined to a single group (*TP53* wild type, n = 77; Additional file 1: Supplementary Table 4. We did not observe any sex specific predominance of *TP53* mutations in contrast to *USP8* mutants that are predominantly found in female patients. Furthermore, we did not find any statistically significant differences in ACTH and cortisol levels (Table2; Additional file 1: Supplementary Table 4).

**Table 2:** Clinical features of *TP53* mutant versus *TP53/USP8* wild type and *USP8* mutant groups

Variable	*TP53* mutant		*TP53/USP8* wild type		*P*-value	*USP8* mutant		*P*-value^a^	
Age at diagnosis (years), mean ± SD	53	± 18	43	± 15	0.069	37	± 13	**0.024**	^b^
Sex (female), n (%)	6/9	(67%)	31/52	(73%)	1.000	25/25	(100%)	** ≥ 0.001**	^c,d^
BMI (kg/m2), mean ± SD	30.6	± 6.0	28.4	± 5.6	0.312	29.0	± 7.8	0.641	
Disease presentation, n (%)					0.065			0.060	
CD	5/9	(56%)	44/52	(85%)		17/25	(68%)		
CTP-BADX/NS	4/9	(44%)	8/52	(15%)		8/25	(32%)		
Number of prior pituitary surgeries, n (%)					**0.009**			**0.034**	^c^
0	2/9	(22%)	31/46	(67%)		17/25	(68%)		
1	4/9	(44%)	13/46	(28%)		6/25	(24%)		
≥ 2	3/9	(33%)	2/46	(4%)		2/25	(8%)		
Total number of pituitary surgeries, n (%)					**0.018**			0.109	
1	2/9	(22%)	29/48	(60%)		15/25	(60%)		
2	3/9	(33%)	14/48	(29%)		6/25	(24%)		
≥ 3	4/9	(44%)	5/48	(10%)		4/25	(16%)		
Complete tumor resection, n (%)	1/8	(13%)	17/28	(61%)	**0.041**	14/17	(82%)	**0.003**	^b^
Postoperative remission, n (%)	4/9	(44%)	28/46	(61%)	0.467	14/23	(61%)	0.670	
Postoperative tumor control, n (%)	4/8	(50%)	14/26	(54%)	1.000	16/23	(70%)	0.494	
Radiation therapy, n (%)	7/9	(78%)	10/38	(26%)	**0.007**	7/23	(30%)	**0.014**	^c^
Radiation therapy before sample collection, n (%)	3/8	(38%)	2/30	(7%)	0.053	2/15	(13%)	0.074	
Bilateral adrenalectomy, n (%)	4/9	(44%)	9/52	(17%)	0.065	10/25	(40%)	**0.048**	
Pharmacological treatments^e^, n (%)	4/6	(67%)	10/23	(44%)	0.390	4/14	(29%)	0.299	
Preoperative hormone levels									
Plasma ACTH (pg/ml), median (IQR)	105	(741.0)	96	(253.4)	0.821	100	(944.4)	0.902	
Serum cortisol (µg/dl), median (range)	29	(19)	23	(2197)	1.000	196	(2469)	0.061	
24 h-urinary free cortisol (µg/24 h), median (range)	1680	(2860)	433	(10,806)	0.674	447	(2735)	0.809	
Serum cortisol after low-dose DST (µg/dl), median (IQR)	8.9	(24.2)	20.0	(14.3)	0.638	31.0	(156.9)	0.217	
Postoperative hormone levels									
Plasma ACTH (pg/mL), median (IQR)	106.0	(216.0)	17.8	(89.5)	0.192	20.0	(78.5)	0.341	
Serum cortisol nadir (µg/dl), median (range)	5.7	(20.8)	7.8	(18.3)	0.907	20.0	(33.3)	0.253	
Disease-specific death, n (%)	3/7	(43%)	1/26	(4%)	**0.023**	1/25	(4%)	**0.010**	

^a^ Adjusted *P*-value for multiple comparisons (*TP53* mutant vs. *TP53/USP8* wild type vs. *USP8* mutant groups)

^b^ *P*  < 0.05 for *TP53* mutant vs. *USP8* mutant groups

^c^ *P*  < 0.05 for *TP53* mutant vs. *TP53/USP8* wild type groups

^d^ *P*  < 0.05 for USP8 mutant vs. *TP53/USP8* wild type groups

^e^ Pharmacological treatments: pasireotide (n = 6), ketoconazole (n = 5), mitotane (n = 5), temozolamide (n = 4) metyrapone (n = 5), cabergoline (n = 3), bevazizumab (n = 1). Five patients received > 1 pharmacological agent

Bold values indicate *P*-values < 0.05

Patients with *TP53* mutant tumors underwent more surgeries and tumor resection was more frequently incomplete compared to *TP53/USP8* wild type (Table 2). These patients also underwent a higher number of additional therapeutic procedures (radiation, n = 7; BADX, n = 4; temozolomide, n = 3; pasireotide, n = 2). Only one patient (#4) with *TP53* mutant tumor, a 77 year-old man, had a single surgery without any other treatment, but his follow-up was short (< 6 months).

We observed *TP53* mutations more frequently in CTP-BADX/NS (4/12, 33%) compared to CD (5/49, 10%), trending towards statistically significant difference (Fischer exact test *P* = 0.065 for *TP53* mutant vs. *TP53/USP8* wild type, *P* = 0.060 for comparison among the 3 groups; Table 2).

**Fig. 1:**
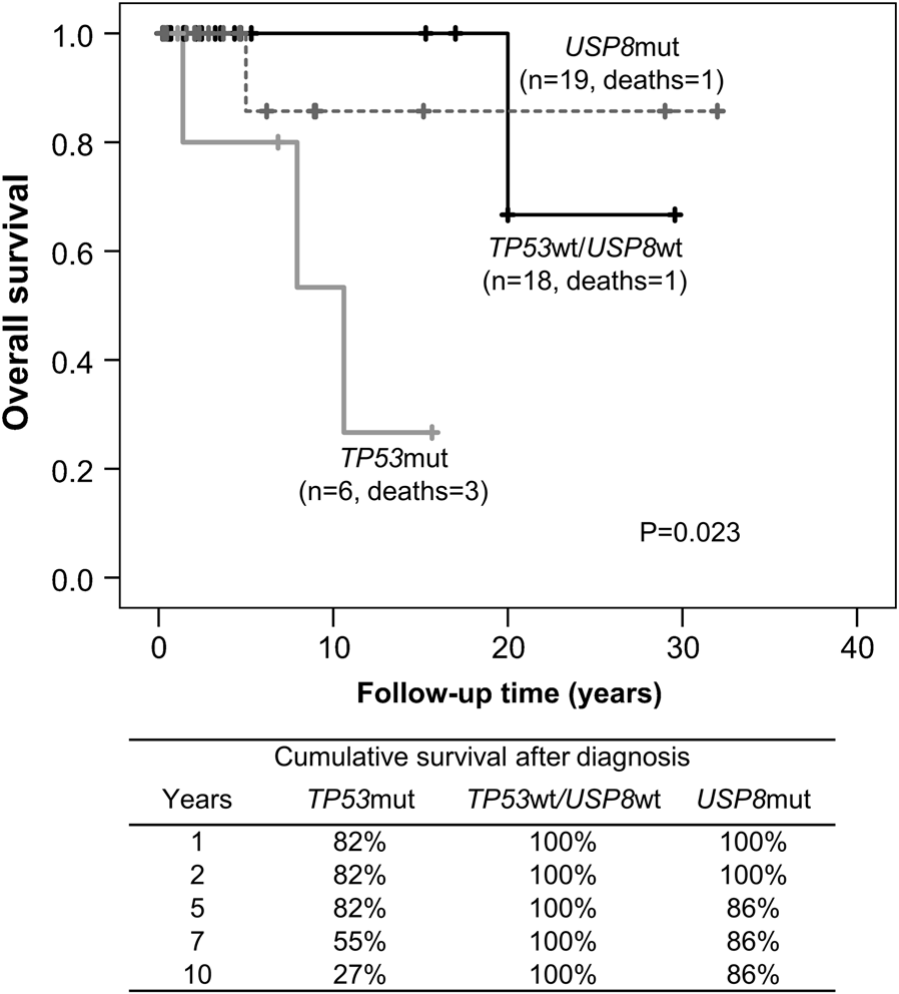
Kaplan–Meier curve showing overall survival in patients with *TP53* mutant/*USP8* wild type, *USP8* mutant/*TP53* wild type, and *TP53* wild type/*USP8* wild type corticotroph tumors. The table underneath the graph shows the 10-year cumulative survival after diagnosis

The *TP53* mutant group associated with higher disease-specific mortality and shorter survival than *USP8* mutant or *TP53/USP8* wild type groups (log rank test, *P* = 0.023, Fig. 1). Three patients with *TP53* mutant tumors (all CTP-BADX/NS) died of disease-related deaths: two from severe cerebral hemorrhage after surgery and stereotactic radiation and one from uncontrolled disease after five failed operations, radiotherapy (gamma knife, fractionated radiation) and chemotherapy (temozolomide, bevacizumab) at the ages of 75, 80 and 37, respectively. Ten-year survival was 27% for patients with *TP53* mutant tumors, 100% for *TP53/USP8* wild type and 86% for *USP8* mutant. In our cohort, survival did not differ after adjusting for age (HR 7.7, 95%CI 0.6–107.7, *P* = 0.127).

Tumor samples from prior surgeries were available from one *TP53* mutant case (#8, Table 1). This male patient had his first pituitary surgery for CD when he was 30 years old and was treated with γ-knife one year later. He then underwent two more pituitary surgeries and BADX until the age of 35. He developed CTP-BADX/NS with para- and retrosellar tumor extension along with panhypopituitarism and underwent two more pituitary surgeries before dying at the age of 38 due to complications of the disease. We detected the *TP53* variant c.1009C > G (p.Arg337Gly) in all available tumor specimens, including his first and latest surgeries (Additional file 1: Supplementary Fig. 1B).

No statistical association was found between clinical data and any of the 8 common variants.

### Characteristics of TP53 mutant corticotroph tumors

All *TP53* mutations were found in macroadenomas (9/66; Table 3). *TP53* mutant tumors were larger that *TP53/USP8* wild type (mm median [IQR] 20.0 [14.0] vs. 15.0 [14.3]), but this did not reach statistical significance (Table 3). Multiple comparison analysis showed that the difference in tumor size is significant only comparing *TP53* mutant with *USP8* mutant (median [IQR] 23.3 [14.0] vs. 14 [7.3] mm; Kruskal–Wallis *P* = 0.019; Bonferroni corrected *P* = 0.018).

**Table 3:** Tumor features of *TP53* mutant versus *TP53/USP8* wild type and *USP8* mutant groups

Variable	*TP53* mutant (n = 9)		*TP53/USP8* wild type (n = 52)		*P*-value	*USP8* mutant (n = 25)		*P*- Value^a^	
Tumor size (mm), median (IQR)	20.0	(14.0)	15.0	(14.3)	0.093	14.0	(7.3)	**0.019**	^b^
Tumor size, n (%)					0.184			0.289	
Microadenoma	0/9	(0%)	13/52	(25%)		6/24	(25%)		
Macroadenoma	9/9	(100%)	39/52	(75%)		18/24	(75%)		
Invasion, n (%)	8/8	(100%)	19/36	(53%)	**0.016**	7/20	(35%)	**0.006**	^b,c^
Hardy grade, n (%)					0.067			**0.029**	^b^
1	0/8	(0%)	7/35	(20%)		6/18	(33%)		
2	1/8	(13%)	13/35	(37%)		8/18	(44%)		
3	3/8	(38%)	11/35	(31%)		4/18	(22%)		
4	4/8	(50%)	4/35	(11%)		0/18	(0%)		
Knosp grade, n (%)					**0.016**			**0.011**	^b,c^
0	0/7	(0%)	2/15	(13%)		3/13	(23%)		
1	1/7	(14%)	6/15	(40%)		5/13	(39%)		
2	0/7	(0%)	1/15	(7%)		2/13	(15%)		
3	0/7	(0%)	4/15	(27%)		3/13	(23%)		
4	6/7	(86%)	2/15	(13%)		0/13	(0%)		
Granulation, n (%)					0.290			0.375	
Sparsely	3/5	(60%)	4/15	(27%)		2/10	(30%)		
Densely	2/5	(40%)	11/15	(73%)		8/35	(70%)		
Ki67 index, median (IQR)	15.0	(14.8)	2.0	(1.0)	**0.006**	2.5	(3.8)	**0.009**	^c^
Ki67 index ≥ 3%, n (%)	5/6	(83%)	3/18	(17%)	**0.007**	6/12	(50%)	**0.008**	^c^
p53 positivity, median (range)	27.5	(41.8)	1.5	(1.0)	0.267	1.0	(1.0)	0.138	

^a^ Adjusted P-value for multiple comparisons (*TP53* mutant vs. *TP53/USP8* wild type vs. *USP8* mutant groups)

^b^ *P* < 0.05 for *TP53* mutant vs. *USP8* mutant groups

^c^ *P* < 0.05 for *TP53* mutant vs. *TP53/USP8* wild type groups

Bold values indicate *P*-values < 0.05

Parasellar invasion was reported in 34 out of 64 cases, for which this information was available, and it was more common in *TP53* mutant tumors (100% vs. 53% and 55% for *TP53/USP8* wild type and *USP8* mutant, respectively; Fischer exact test *P* = 0.006). *TP53* mutant tumors had higher Knosp grade (Kruskal–Wallis *P* = 0.011) with the majority being Knosp 4 (Table 3, Additional file 1: Supplementary Table 4).

Ki67 proliferation index was available for 36 cases (6 *TP53* mutant). Five out of six *TP53* mutant tumors had Ki67 ≥ 3% and the overall Ki67 was higher than in the wild type tumors (Kruskal–Wallis *P* = 0.01; Bonferroni corrected *P* = 0.008 for *TP53/USP8* wild type) (Table 3). Ki67 ≥ 10% was reported in 6 tumors, from which 5 were *TP53* mutant (Fischer exact test *P* < 0.0001; the remaining case was *TP53/USP8* wild type).

We had information on p53 immunostaining from 9 cases (all macroadenomas), four of which *TP53* mutant: 3 tumors (from patients #5, 6 and 9) showed high p53 immunoreactivity, while the one (from patient #3) carrying a nonsense variant leading to a truncated protein was p53 negative. The five *TP53* wild type cases showed isolated nuclear staining in < 1–3% of cells.

Summarizing, *TP53* mutations were significantly associated with features related to a more aggressive tumor behavior, such as incomplete tumor resection, more frequent parasellar invasion, higher Knosp grade, and higher Ki67 proliferation index (Table 3; Additional file 1: Supplementary Table 4).

## Discussion

Herein, we investigated the prevalence of *TP53* mutations by screening a large cohort of 61 functional corticotroph tumors with *USP8* wild type status, and found variants altering protein function in 15% of cases. We did not detect *TP53* mutations in a separate group of 25 *USP8* mutant tumors, which is in concordance with previously published small next-generation sequencing series [8, 18, 19].

Since we focused on *USP8* wild type tumors, macroadenomas were overrepresented in our cohort. Consequently, it should be noted that the prevalence of *TP53* mutations is expected to be lower in the general CD population. In fact, ~ 50% of corticotroph tumors carry *USP8* mutations, which others and we have shown to be mutually exclusive. Corticotroph tumors with *USP8* mutations are associated with female predominance, younger age at presentation, and less invasiveness (despite shorter time to relapse) [9, 11, 13, 18, 36]. In contrast, *TP53* mutant tumors were diagnosed mostly at older age, did not show sex predominance and were larger and more invasive, with lower complete resection rate. None of the 19 microadenomas included in our study carried *TP53* mutations. Still, we need to acknowledge that since no sample was microdissected we may have lost microadenoma cases with *TP53* mutations. Instead, we found *TP53* mutations in 9/66 macroadenomas (14%) and 8/34 (24%) invasive tumors, supporting the findings from smaller series [17, 19].

Tumor size at presentation or invasiveness do not reliably predict aggressiveness. Instead, the European Society of Endocrinology Clinical Practice Guidelines for the management of aggressive pituitary tumors and carcinomas proposed a definition of pituitary tumor aggressiveness based on rapid or clinically relevant tumor growth despite optimal therapeutic options, along with bone invasion [37]. A recent study in a series of 9 aggressive pituitary tumors and carcinomas carrying *ATRX* mutations reported a high frequency of missense *TP53* variants (5/9, 55.6%), further suggesting a link between *TP53* mutational status and unfavorable outcome [20]. We do not have exact information on changes of tumor growth for the majority of our cases, but the higher number of surgical and radiation interventions, the higher Knosp grades, and the increased mortality rate indicate that patients with *TP53* mutant tumors obviously follow a more aggressive disease course.

Ki67 proliferation index together with p53 immunostaining and mitotic count have been suggested as histological markers of pituitary tumor aggressiveness [29, 38]. In our series, Ki67 was significantly higher in *TP53* mutant tumors, reinforcing our prior observation of a higher proportion of *TP53* mutant tumors in the Ki67 ≥ 3 group [17]. We had limited information on p53 immunohistochemistry, since this measure is not routinely performed in our collaborative centers. Nevertheless, in the few tumors with known p53 immunopositivity, it was higher in the *TP53* mutant group, which is in concordance with a previous study reporting high p53 immunoreactivity in all *TP53* mutant tumors [19].

A mutagenic action of radiation on *TP53* has been hypothesized by small series on radiation-induced tumors. For instance, *TP53* mutations were reported in 58% of radiation-induced sarcomas [39], while a meta-analysis reported *TP53* mutations in 14/30 radiation-induced gliomas [40]. A previous study reported a case with frameshift *TP53* mutation in the CTP-BADX/NS tumor, but not in the initial CD surgeries, and the mutation was therefore suspected to be induced by radiotherapy [41]. In our series, however, 4 out of 7 *TP53* mutant tumors were obtained before radiation.

In their case report, Pinto et al*.* suggested that *TP53* mutations are acquired during tumorigenesis and condition tumor evolution [41]. In contrast, Casar-Borota et al*.* and Uzilov et al. reported high allele fraction of *TP53* mutations, indicating that they are not a late event in corticotroph tumorigenesis [19, 20]. In addition, Uzilov et al*.* reported *TP53* mutations in all tumor specimens from their two *TP53* mutant cases with multiple surgeries [19]. Similarly, in our series we had tissue from multiple pituitary surgeries from one patient and found the *TP53* variant in all samples (CD and CTP-BADX/NS), including specimens obtained before radiotherapy. Taken together, these observations suggest that in most cases, *TP53* mutations may appear early during tumor development.

A limitation of our study is the short follow-up of patients who were prospectively included. Moreover, material from repeated surgeries was lacking from most patients with *TP53* mutant tumors, hampering the examination of tumor evolution in these patients. Similarly, we had limited access to blood samples, so we could not demonstrate the somatic origin for all variants. Nevertheless, the older age at initial diagnosis of CD in patients with *TP53* mutant tumors (53 ± 19.5 years old, with the youngest patient diagnosed at the age of 30) and the absence of additional neoplasias during follow-up also support a somatic instead of a germline origin. Furthermore, conditions related to germline *TP53* mutations, such as Li-Fraumeni syndrome, very rarely present with pituitary tumor [42]. To our knowledge, the only published case so far was a pediatric patient with an aggressive lactotroph tumor [43].

In addition to the *TP53* mutations, we detected several common variants. Variants rs59758982 and rs1042522 have been associated with increased cancer susceptibility [44, 45]. In some cancer types, the very frequent rs1042522 c.215G > C (p.Pro72Arg) alternative variant correlated to more efficient induction of apoptosis by DNA-damaging chemotherapeutic drugs, growth suppression and higher metastatic potential [46–48]. In nonfunctioning pituitary tumors, alternative allele C (leading to p.Arg72) was related to early age at presentation and reduced p21 expression [49]. Very recently, an overrepresentation of the rs1042522 alternative allele C (p.Arg72) was reported in 9 out of 10 corticotroph neoplasias including 5 functional tumors (allele frequency 0.900, vs 0.714 in Latino/admixed American in gnomAD [31]) without any association with clinical features [50]. In our cohort, we did not detect different allele frequencies in any of the investigated common variants (including rs1042522) compared with public databases, nor statistical association with any clinical variable, rendering their contribution to corticotroph pathophysiology unlikely.

## Conclusion

Screening a large corticotroph tumor series revealed that *TP53* mutations are more frequent than previously considered. Furthermore, we show that patients with *TP53* mutant tumors had higher number of surgeries, more invasive tumors, and worse disease outcome. Our study provides evidence that patients with pathogenic or function altering variants may require more intense treatment and extended follow-up, and suggests screening for *TP53* variants in macroadenomas with wild type *USP8* status. Further work is needed to determine the potential use of *TP53* status as a predictor of disease outcome.

## Supplementary Information

**Additional file 1**. **Supplementary Table 1**: Description of study cohort. **Supplementary Table 2**: Primers used for *TP53* amplification and Sanger sequencing. **Supplementary Table 3**: Common *TP53* variants in the study cohort. **Supplementary Table 4:** Comparison of *TP53 *mutant versus *TP53* wild type group. **Supplementary Figure 1**. Chromatograms showing the *TP53 *variants found in the corticotroph tumor of patient #1 and #8 (Table 1). A. The variant c.398T>A was present in homozygocity in the tumor and absent in the blood. B. The variant c.1009C>G is detected in all available surgical specimens in this patient. First and 2nd surgeries were Cushing’s disease tumors and 4th and 5th CTP-BADX/NS.

## Data Availability

The authors declare that the relevant data supporting the conclusions of this article are included within the article and its supplementary information file. Additional clinical data are available from the corresponding authors MT and LGPR upon reasonable request.

## Abbreviations

CD: Cushing’s disease
BADX: Bilateral adrenalectomy
CTP-BADX/NS: Corticotroph tumor progression after bilateral adrenalectomy/Nelson’s syndrome
ACTH: Adrenocorticotropic hormone
SD: Standard deviation
IQR: Interquartile range
HR: Hazard ratio
