# Supplementary material for: TP53 mutations in functional corticotroph tumors are linked to invasion and worse clinical outcome
Source: Acta Neuropathol Commun. 2022 Sep 19;10:139. doi: 10.1186/s40478-022-01437-1 (PMC9484083; doi:10.1186/s40478-022-01437-1)
Supplement: Supplementary file 1 — Additional file 1. Supplementary Table 1: Description of study cohort. Supplementary Table 2: Primers used for TP53 amplification and Sanger sequencing. Supplementary Table 3: Common TP53 variants in the study cohort. Supplementary Table 4: Comparison of TP53 mutant versus TP53 wild type group. Supplementary Figure 1. Chromatograms showing the TP53 variants found in the corticotroph tumor of patient #1 and #8 (Table 1). A. The variant c.398T>A was present in homozygocity in the tumor and absent in the blood. B. The variant c.1009C>G is detected in all available surgical specimens in this patient. First and 2nd surgeries were Cushing’s disease tumors and 4th and 5th CTP-BADX/NS. [file 40478_2022_1437_MOESM1_ESM.pdf]

Supplementary Table 1. Description of study cohort.

Variable	mean/median	SD/IQR	Total n
Age at diagnosis (years), mean ±SD, [total n]	42	±15.2	86
Sex (female), n (%), [total n]	62	(72%)	86
BMI (kg/m2), mean ±SD, [total n]	28.9	±6.3	74
Disease presentation, n (%), [total n]			86
Cushing	66	(77%)	
Nelson	20	(23%)	
Number of prior pituitary surgeries, n (%), [total n]			80
0	50	(63%)	
1	23	(29%)	
≥2	7	(9%)	
Total number of pituitary surgeries, n (%), [total n]			82
1	46	(56%)	
2	23	(28%)	
≥3	13	(16%)	
Complete tumor resection, n (%), [total n]	32	(60%)	53
Postoperative remission, n (%), [total n]	46	(59%)	78
Postoperative tumor control, n (%), [total n]	34	(60%)	57
Radiation therapy, n (%), [total n]	24	(34%)	70
Radiation therapy before sample collection, n (%), [total n]	7	(13%)	53
Bilateral adrenalectomy, n (%), [total n]	23	(27%)	86
Pharmacological treatments[a], n (%), [total n]	18	(42%)	43
Preoperative hormone levels			
Plasma ACTH (pg/mL), median (IQR)	98	(570.4)	75
Serum cortisol (μg/dl), median (range)	29.1	(168.6)	50
24h-urinary free cortisol (μg/24h), median (range)	432.5	(598.3)	30
Serum cortisol after low-dose DST (μg/dl), median (IQR)	20	(20.7)	46
Postoperative hormone levels			
Plasma ACTH (pg/mL), median (IQR)	20	(107.6)	57
Serum cortisol nadir (μg/dl), median (range)	8.8	(19.4)	58
Tumor size (mm), median (IQR), [total n]	15	(13.0)	85
Microadenoma	19	(22%)	
Macroadenoma	66	(78%)	
Granulation, n (%), [total n]			30
Sparsely	9	(30%)	
Densely	21	(70%)	
Ki67 index, median (IQR), [total n]	2.0	(3.8)	36
Ki67 index ≥3%, n (%)	14	(39%)	36
p53 positivity, median (IQR), [total n]	1	(26.5)	9
Invasion, n (%), [total n]	34	(53%)	64
Hardy grade, n (%), [total n]			61
1	13	(21%)	
2	22	(36%)	
3	18	(30%)	
4	8	(13%)	
Knosp grade, n (%), [total n]			35
0	5	(14%)	
1	12	(34%)	
2	3	(9%)	
3	7	(20%)	
4	8	(7%)	
Disease-specific death, n (%), [total n]	5	(9%)	58

[a] Pharmacological treatments: pasireotide (n=6), ketoconazole (n=5), mitotane (n=5), temozolamide (n=4) metyrapone (n=5), cabergoline (n=3), bevazizumab (n=1). Five patients received >1 pharmacological agent.

Supplementary Table 2. Primers used for *TP53* amplification and Sanger sequencing.

Primer	Sequence	DNA source
TP53-1	5'-TCTCATGCTGGATCCCCACT-3'	FF, FFPE
TP53-1rv	5'-GACCAGGTCCTCAGCC-3'	FFPE
TP53-2fw	5'-GGGGGCTGAGGACCTGGT-3'	FFPE
TP53-2rv	5'-ATACGGCCAGGCATTGAAGT-3'	FFPE
TP53-2	5'-AGAGGAATCCCAAAGTTCCA-3'	FF
TP53-3	5'-GTGCCCTGACTTTCAACTC-3'	FF, FFPE
TP53-3rv	5'-GGCAACCAGCCCTGTC-3'	FFPE
TP53-4fw	5'-GCCTCTGATTCCTCACTGAT-3'	FFPE
TP53-4	5'-CAGGAGAAAGCCCCCCTACT-3'	FF, FFPE
TP53-5	5'-CTTGCCACAGGTCTCCCCAA-3'	FF, FFPE
TP53-6	5'-AGGGGTCAGAGGCAAGCAGA-3'	FF, FFPE
TP53-7	5'-TAGGACCTGATTTCCTTA-3'	FF, FFPE
TP53-7rv	5'-AGTGAATCTGAGGCATAAC-3'	FFPE
TP53-7Bfw	5'-TGGAGGAGACCAAGGGTG-3'	FFPE
TP53-7Brv	5'-CGGCATTTTGAGTGTTAGAC-3'	FFPE
TP53-8	5'-TAAGCTATGATGTTCCTTAG-3'	FF, FFPE
TP53-8rv	5'-GACTGTTTTACCTGCAATTG-3'	FFPE
TP53-9	5'-CAATTGTAACTTGAACCATC-3'	FF, FFPE
TP53-10	5'-GGATGAGAATGGAATCCTAT-3'	FF, FFPE
TP53-11	5'-TCTCACTCATGTGATGTCATC-3'	FF, FFPE
TP53-12	5'-CACACCTATTGCAAGCAAGG-3'	FF, FFPE

FF, fresh frozen; FFPE, formalin-fixed paraffin embedded.

Supplementary Table 3. Common *TP53* variants in the study cohort.

SNP ID	Variant in chromosome 17 (GRCh38/hg38)	Present study		ALFA[a] (version 20201027095038)			gnomAD[b] (v3.1.2, genomes)			1000 Genomes Project[a] (phase 3)		
		Allele frequency	Count	Allele frequency	Count	P-value[c]	Allele frequency	Count	P-value[c]	Allele frequency	Count	P-value[c]
rs1800370	7676261					0.603			1.000			1.000
	C	0.986	71	0.987	54859		0.986	63667		0.983	989	
	T	0.014	1	0.013	707		0.014	905		0.017	17	
rs1042522	7676154					0.708			0.613			1.000
	G	0.280	23	0.263	23805		0.256	16523		0.285	287	
	C	0.720	59	0.737	66583		0.744	48001		0.715	719	
rs1642785	7676483					0.692			0.496			0.892
	G	0.300	21	0.281	5733		0.261	16851		0.288	290	
	A	0.700	49	0.719	14665		0.739	47621		0.712	716	
rs540683791	7676324					0.072			-			0.187
	C	0.986	71	0.999	9629		NA	NA		0.998	1004	
	G	0.014	1	0.001	9		NA	NA		0.002	2	
rs59758982	7676335_7676366					0.634			1.000			<0.001[d]
	(CCCCAGCCCTCC AGGT)(C)$_4$AGCCC	0.141	10	0.167	886		0.146	9404		0.378	380	
	(CCCCAGCCCTCC AGGT)$_2$(C)$_4$AGCCC	0.859	61	0.833	4414		0.854	54872		0.622	626	
rs17883323	7676301					0.800			0.799			0.793
	G	0.965	70	0.942	34434		0.946	61022		0.942	948	
	T	0.035	3	0.058	2122		0.054	3510		0.058	58	
rs1625895	7674797					0.341			0.419			0.254
	T	0.089	8	0.135	29617		0.128	8250		0.143	144	
	C	0.911	77	0.865	189339		0.872	56295		0.857	862	

[a] European cohort only

[b] Non-Finnish European cohort only

[c] Exact two sided P-value for comparison with the present study (Chi-square test).

[d] rs59758982 allele frequencies differ in 1KG from all other databases.

Supplementary Table 4. Comparison of *TP53* mutant versus *TP53* wild type group.

Variable	*TP53* mutant (n=9)		*TP53* Wild-type (n=77)		P-value
Age at diagnosis (years), mean ±SD	53	±18	41	±21	**0.023**
Sex (female), n (%)	6/9	(67%)	56/77	(73%)	0.705
BMI (kg/m2), mean ±SD	30.6	±6.0	28.7	±6.4	0.412
Disease presentation, n (%)					0.203
CD	5/9	(56%)	61/77	(79%)	
CTP-BADX/NS	4/9	(44%)	16/77	(21%)	
Number of prior surgeries, n (%)					**0.007**
0	2/9	(22%)	48/71	(67%)	
1	4/9	(44%)	19/71	(27%)	
≥2	3/9	(33%)	4/71	(6%)	
Total number of surgeries, n (%)					**0.021**
1	2/9	(22%)	44/73	(60%)	
2	3/9	(33%)	20/73	(28%)	
≥3	4/9	(44%)	9/73	(12%)	
Complete tumor resection, n (%)	1/8	(13%)	31/45	(69%)	**0.001**
Postoperative remission, n (%)	4/9	(44%)	42/69	(61%)	0.475
Postoperative tumor control, n (%)	4/8	(50%)	30/49	(61%)	0.429
Radiation therapy, n (%)	7/9	(78%)	17/61	(28%)	**0.014**
Radiation therapy before sample collection, n (%)	3/8	(38%)	4/5	(9%)	**0.043**
Bilateral adrenalectomy, n (%)	4/9	(44%)	19/76	(25%)	**0.240**
Pharmacological treatments, n (%)	4/6	(67%)	14/37	(38%)	0.379
Preoperative hormone levels					
Plasma ACTH (pg/mL), median (IQR)	105	(741)	98	(448)	0.855
Serum cortisol (µg/dl), median (range)	29	(19)	29	(175)	0.753
24h-urinary free cortisol (µg/24h), median (range)	1680	(2860)	433	(591)	0.607
Serum cortisol after low-dose DST (µg/dl), median (IQR)	8.9	(24.2)	20.0	(20.3)	0.439
Postoperative hormone levels					
Plasma ACTH (pg/mL), median (IQR)	106.0	(216.0)	19.0	(75.3)	0.225
Serum cortisol nadir (µg/dl), median (range)	5.7	(20.8)	9.0	(20.2)	0.857
Tumor size (mm), median (IQR)	20.0	(14.0)	15.0	(12.1)	**0.026**
Tumor size, n (%)					0.194
Microadenoma	0/9	(0%)	19/76	(25%)	
Macroadenoma	9/9	(100%)	57/76	(75%)	
Granulation, n (%)					0.188
Sparsely	3/5	(60%)	6/25	(24%)	
Densely	2/5	(40%)	19/25	(76%)	
Ki67 index, median (IQR)	15.0	(14.8)	2.0	(2.0)	**0.014**
Ki67 index ≥3%, n (%)	5/6	(83%)	9/30	(28%)	**0.024**
p53 positivity, median (IQR)	27.5	(41.8)	1.0	(0.5)	0.111
Invasion, n (%)	8/8	(100%)	26/56	(46%)	**0.011**
Hardy grade, n (%)					**0.001**
1	0/8	(0%)	13/53	(24%)	
2	1/8	(13%)	21/53	(40%)	
3	3/8	(38%)	15/53	(28%)	
4	4/8	(50%)	4/53	(8%)	
Knosp grade, n (%)					**≤0.001**
0	0/7	(0%)	5/28	(18%)	
1	1/7	(14%)	11/28	(39%)	
2	0/7	(0%)	3/28	(11%)	
3	0/7	(0%)	7/28	(25%)	
4	6/7	(86%)	2/28	(7%)	
Disease-specific death, n (%)	3/7	(43%)	2/51	(4%)	**0.006**

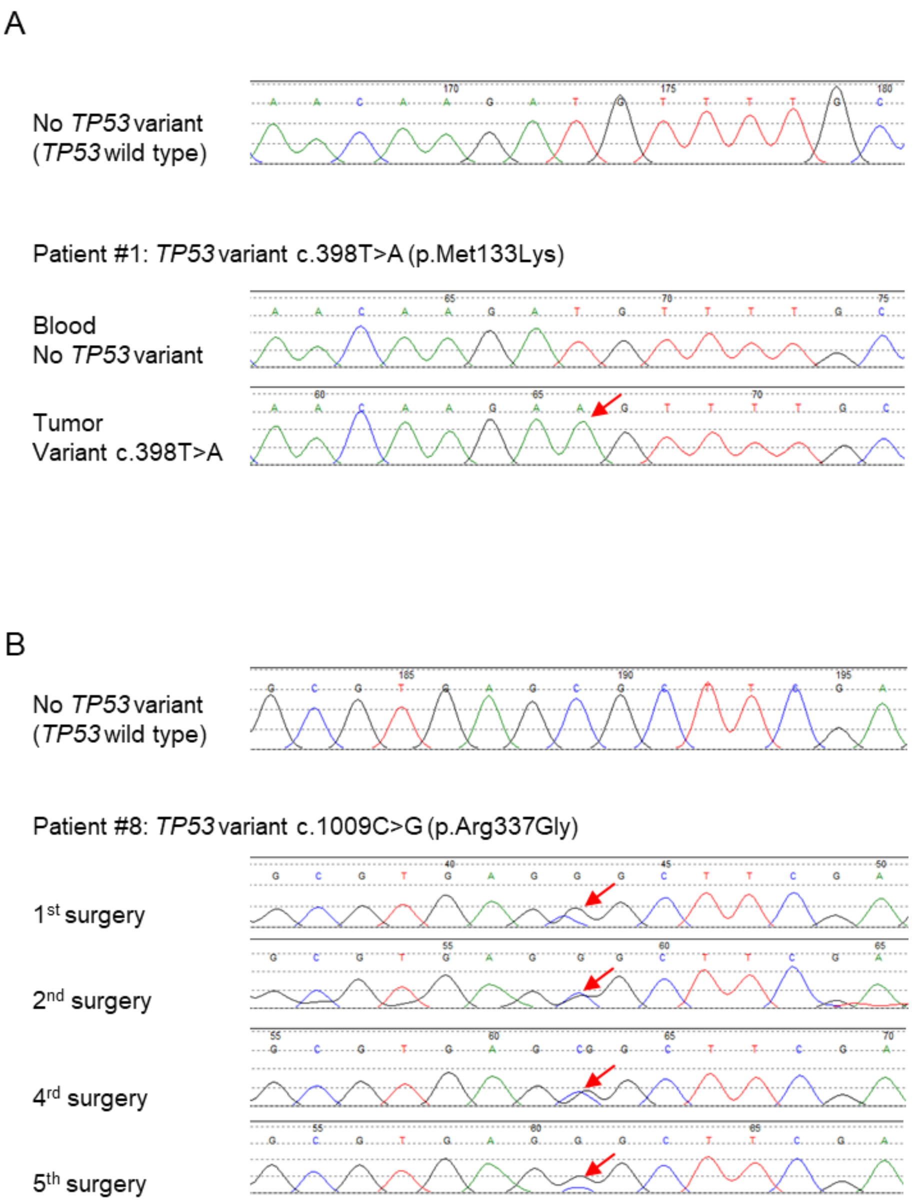

Supplementary Figure 1. Chromatograms showing the TP53 variants found in the corticotroph tumor of patient #1 and #8 (Table 1). A. The variant c.398T>A was present in homozygocity in the tumor and absent in the blood. B. The variant c.1009C>G is detected in all available surgical specimens in this patient. First and 2nd surgeries were Cushing's disease tumors and 4th and 5th CTP-BADX/NS.
